# Risk factors of recurrence after drug withdrawal in children with epilepsy

**DOI:** 10.3389/fneur.2023.1122827

**Published:** 2023-04-27

**Authors:** Yongheng Zhao, Hao Ding, Xiaoyu Zhao, Xiaochang Qiu, Baomin Li

**Affiliations:** ^1^Department of Pediatrics, Qilu Hospital of Shandong University, Jinan, China; ^2^Cheeloo College of Medicine, Shandong University, Jinan, China; ^3^Department of Geriatrics, The First Affiliated Hospital of Shandong First Medical University (Shandong Provincial Qianfoshan Hospital), Jinan, China

**Keywords:** epilepsy, children, drug withdrawal, recurrence, risk factors

## Abstract

This study aimed to evaluate the risk factors for recurrence in pediatric patients with epilepsy following normal antiseizure medication (ASM) treatment and drug withdrawal. We retrospectively analyzed 80 pediatric patients who received treatment at the Qilu Hospital of Shandong University between January 2009 and December 2019 after at least 2 years of seizure-free and normal electroencephalography (EEG) before the regular drug reduction. Patients were followed-up for at least 2 years and divided into the recurrence and nonrecurrence groups based on whether relapse occurred. Clinical information was gathered, and the risk variables for recurrence were statistically analyzed. Post 2 years of drug withdrawal, 19 patients showed relapses. The recurrence rate was 23.75%, and the mean time of recurrence was 11.09 ± 7.57 months, where 7 (36.8%) were women and 12 (63.2%) were men. In all, 41 pediatric patients were followed-up until the 3rd year, of which 2 (4.9%) patients experienced a relapse. Among the remaining 39 patients without relapse, 24 were followed-up until the 4th year, and no recurrence occurred. After being monitored for >4 years, 13 patients experienced no recurrence. The differences in the history of febrile seizures, combined use of ≥2 ASMs, and EEG abnormalities after drug withdrawal between the two groups were statistically significant (*p* < 0.05). Multivariate binary logistic regression analysis revealed that these factors are independent risk factors for recurrence after drug withdrawal in children with epilepsy: history of febrile seizures (OR = 4.322, 95% CI: 1.262–14.804), combined ASM use (OR = 4.783, 95% CI: 1.409–16.238), and EEG abnormalities after drug withdrawal (OR = 4.688, 95% CI: 1.154–19.050). In summary, our results suggest that the probability of seizure recurrence following drug cessation may be greatly increased by a history of febrile seizures, concomitant use of ≥2 ASMs, and EEG abnormalities after drug cessation. The majority of recurrences occurred in the first 2 years following drug discontinuation, whereas the rate of recurrence was minimal thereafter.

## 1. Introduction

Epilepsy is a chronic, recurrent paroxysmal disease characterized by highly synchronized abnormal discharge of neurons in the brain (1). Epilepsy occurs 0.7% of the time in international epidemiological surveys, and children make up two-thirds of all cases (2). The pathogenesis of epilepsy is complex and not entirely understood. Simultaneously, the most widely recognized cause is an imbalance between central nervous system excitation and inhibition (3), which may be closely related to neurotransmitters, ion channels (4, 5), and cytokines (6). Epilepsy can cause varying degrees of motor function and intellectual impairment and can affect growth and development. As a result, pediatric epilepsy has been widely concerned and is the focus of epilepsy control in the entire population. Currently, the primary treatment is medication. Seizures are managed in 80% of juvenile patients, the prognosis is good, the possibility of recurrence is remarkably decreased, and the patients have normal social adaptation skills; however, the risk of recurrence after drug withdrawal still remains. Refractory epilepsy affects 20–40% of pediatric patients who have uncontrolled seizures and a dismal prognosis (7, 8). Even if long-term pharmacological use will promote seizure control, the pediatric patient and family will consequently bear a heavy physical and psychological burden. Therefore, when to start drug cessation has become an important problem for pediatricians. However, the standard for drug withdrawal remains unclear. The currently recognized practice is the cessation of drugs when a patient has been in clinical remission for >2 years, and in adults, this time frame is extended to 3–4 years (9, 10). The greatest risk of drug withdrawal is epilepsy recurrence or even the development of drug-resistant seizures. By following up juvenile patients who had been seizure-free for >2 years, some researchers discovered that 28.6% of the patients reverted during tapering or after drug withdrawal, with recurrences predominating in the first 2 years after drug withdrawal. After >2 years of drug withdrawal, the likelihood of recurrence is reduced (11–13). Because the risk factors for recurrence after epilepsy drug withdrawal are debatable, the early identification of risk factors can help determine the patient’s risk of relapse and help clinicians decide whether and when to discontinue the medication. Therefore, the identification of risk factors for recurrence and appropriate intervention after drug withdrawal are important.

## 2. Materials and methods

### 2.1. General information

In all, 80 pediatric patients who underwent treatment at the Pediatric Neurology Department of Shandong University, Qilu Hospital, between January 2009 and December 2019 with a proposed reduction of antiseizure medications (ASMs), were included as study participants. The age of epilepsy onset ranged between 0 and 14 years. The inclusion criteria were as follows: (1) The pediatric patients’ epilepsy diagnosis met the 2014 epilepsy definition of the International League Against Epilepsy (1). (2) All children could adhere to the doctor’s advice for oral drug therapy and could complete brain magnetic resonance imaging, computed tomography, electroencephalography (EEG), and other related examinations on time. (3) The juvenile patient underwent drug withdrawal when it had been >2 years since the last seizure, the EEG was normal, and the duration of drug withdrawal had exceeded 6 months. (4) The follow-up period after complete drug withdrawal was 2 years.

### 2.2. Study methods

The pediatric patients underwent routine diagnosis and treatment, did not experience a seizure for ≥2 years in a row, had EEG findings that returned to normal, and tapered for 6 months before beginning drug withdrawal. This was implemented after a thorough evaluation and with the patient’s and their guardian’s consent. Outpatient or telephone follow-up was conducted once every 3 months after drug withdrawal, and all patients had completed 2 years of follow-up. Detailed information about the pediatric patients was recorded, including birthdate, sex, onset age, family history, pretreatment seizure frequency, pretreatment disease course, age upon drug withdrawal, seizure manifestations, EEG results at various stages, time of drug withdrawal, and recurrence. EEG abnormalities include both epileptiform discharges and slowing. Relevant factors were selected by consulting the relevant literature at home and abroad, combined with the clinical experience and follow-up data of the team.

### 2.3. Statistical methods

The statistical analysis was performed using IBM SPSS Statistics for Windows version 26.0 (IBM Corp., Armonk, NY, United States). The mean ± standard deviation was used to convey normally distributed quantitative data. The *χ*^2^ test was used to analyze qualitative data. Relevant factors were examined using multivariate binary logistic regression analysis, and a difference with *p* < 0.05 was deemed statistically significant.

## 3. Results

### 3.1. Recurrence after drug withdrawal

In total, 80 pediatric patients discontinued drug therapy, of whom 35 (43.8%) were girls and 45 (56.3%) were boys. All pediatric patients were followed-up until 2 years after drug withdrawal. During this period, 19 recurrences occurred, and 61 cases did not relapse. These patients were split into two groups: those who experienced recurrence and those who did not. The overall recurrence rate was 23.8%, with 12 male and 7 female patients having a relapse, accounting for 63.2 and 36.8% of the total number of relapsed cases, respectively.

### 3.2. Correlation between the time of drug withdrawal and recurrence rate

**Figure 1:**
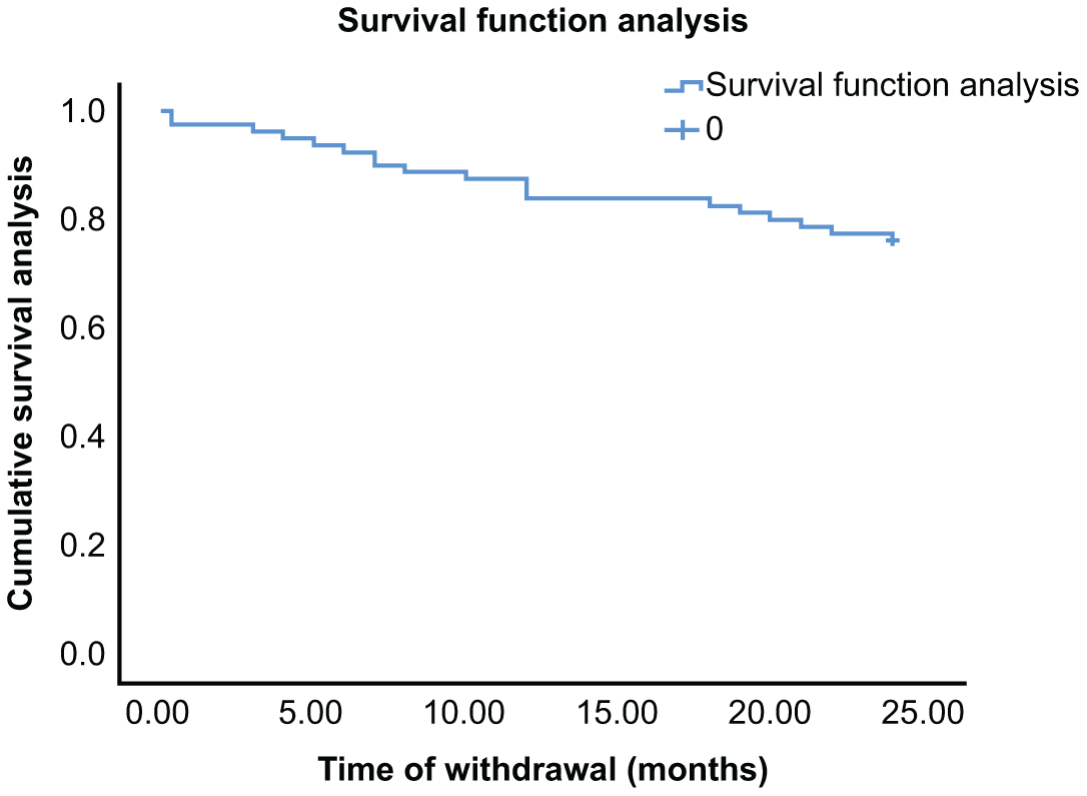
Kaplan–Meir survival curve relationship between the time of drug withdrawal and nonepilepsy recurrence rate in all pediatric patients. Zero represents the time of drug withdrawal. The abscissa of the curve represents the time after drug withdrawal, whereas the ordinate represents the proportion of the total number of patients without relapse.

In all, 61 pediatric patients who underwent drug withdrawal did not relapse in the first 2 years of follow-up. Among them, 53 patients received follow-up care for >2 years; however, only 41 of them completed the third year of follow-up because of loss to follow-up and other reasons, and 2 (4.9%) patients had a relapse. Of the remaining 39 non-relapse patients, 24 were monitored through the fourth year without experiencing a relapse. Moreover, 13 of these 24 patients were followed-up for >4 years and did not have a recurrence. The longest follow-up duration was 7 years. Thus, relapse after drug withdrawal was mainly concentrated in the first 2 years after drug withdrawal, and the recurrence rate was low after 2 years (Table 1). Owing to the small sample size during follow-up, the conclusions are limited. The survival curve shows that, in the 2 years following drug withdrawal, 6 (7.5%), 13 (16.3%), and 14 (17.5%) patients relapsed during the first 6, 12, and 18 months, respectively (Figure 1). Briefly, the curve indicates that the proportion of people without relapse after drug withdrawal in the total population decreases with time.

**Table 1:** Correlation between the drug withdrawal time and recurrence rate in pediatric patients with epilepsy.

Follow-up time	Total number of patients	Number of relapsed patients	Number of non-relapse patients
		*n* (%)	*n*
Followed-up for 2 years	80	19 (23.8)	61
3rd year of follow-up	41	2 (4.9)	39
4th year of follow-up	24	0	24
Followed-up for >4 years	13	0	13

### 3.3. Comparison of general information between the two groups

**Figure 2:**
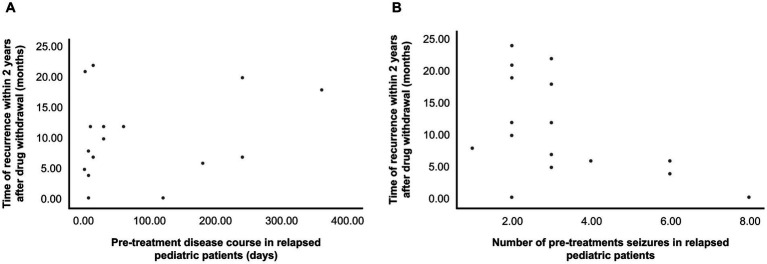
**(A)** Relationship between pretreatment disease course and time of postwithdrawal recurrence in the relapse group. Three points with high deviation were excluded from the figure. **(B)** Relationship between pretreatment seizure frequency and time of postwithdrawal recurrence in the relapse group. Nineteen patients had a relapse. In the figure, an overlap occurs between two pediatric patients, and two points of high deviation were excluded.

Sex, family history, age of onset, pretreatment seizure frequency, pretreatment illness course, age upon drug withdrawal, pretreatment EEG, and seizure type did not statistically differ significantly between the two groups (*p* > 0.05). Compared with the nonrecurrence group, the recurrence group had a significantly higher number of pediatric patients with a history of febrile seizures, combination therapy with ≥2 ASMs, and EEG abnormalities following drug withdrawal (*p* < 0.05) (Table 2). The recurrence rates in pediatric patients with an age of onset of 0–3 and ≥ 4 years were 27.7 and 19.2%, respectively, which were higher than the recurrence rate (14.3%) in pediatric patients aged 3–4 years (Table 3). In the children who relapsed after drug withdrawal, no regularity was found in the scatter plot between the course of disease before treatment, the number of seizures, and the time of relapse after drug withdrawal (Figure 2).

**Table 2:** Univariate analysis of pediatric epilepsy relapse 2 years after drug withdrawal.

Item	Recurrence group	Nonrecurrence group	*χ*^2^ value	*p*-value
	(n = 19)	(n = 61)		
Sex [n (%)]				
Male	12 (63.2)	33 (54.1)	0.483	0.487
Female	7 (36.8)	28 (45.9)		
Family history				
Yes	4 (21.1)	8 (13.1)	0.716	0.397
No	15 (78.9)	53 (86.9)		
History of febrile seizures				
Yes	12 (63.2)	21 (34.4)	4.935	0.026
No	7 (36.8)	40 (65.6)		
Pretreatment seizure frequency				
>1 seizure/month	12 (63.2)	41 (67.2)	0.107	0.744
≤1 seizure/month	7 (36.8)	20 (32.8)		
Pretreatment disease course				
≤1 year	16 (84.2)	57 (93.4)	0.606	0.436
>1 year	3 (15.8)	4 (6.6)		
Combined ASM treatment				
Yes	11 (57.9)	17 (27.9)	5.741	0.017
No	8 (42.1)	44 (72.1)		
Age of drug withdrawal				
≤10 years	15 (78.9)	44 (72.1)	0.085	0.771
>10 years	4 (21.1)	17 (27.9)		
EEGs during confirmed epilepsy				
Normal	2 (10.5)	18 (29.5)	1.864	0.172
Abnormal	17(89.5)	43 (70.5)		
EEG monitoring 6 months after drug withdrawal				
Normal	13 (68.4)	56 (91.8)	4.853	0.028
Abnormal	6 (31.6)	5 (8.2)		
Seizure type				
Focal seizure Generalized seizure Focal secondary generalized seizure	9 (47.4) 10 (52.6) 0 (0)	18 (29.5) 37 (60.7) 6 (9.8)	2.918	0.222

**Table 3:** Comparison of age of onset between the two groups.

Age of onset	Recurrence group	Nonrecurrence group	*χ*^2^ value	*p*-value
	*n* (%)	*n* (%)		
<3 years	13 (27.7%)	34 (72.3%)		
3–4 years	1 (14.3%)	6 (85.7%)	1.036	0.596
>4 years	5 (19.2)	21 (80.8%)		

### 3.4. Multivariate analysis of recurrence after drug withdrawal

Markers with statistically significant differences in the univariate analysis results were used for the multivariate binary logistic regression analysis. The findings revealed that EEG abnormalities following drug withdrawal (OR = 4.688, 95% CI: 1.154–19.050), concomitant ASM therapy (OR = 4.783, 95% CI: 1.409–16.238), and a history of febrile seizures (OR = 4.322, 95% CI: 1.262–14.804) were independent risk factors for recurrence following drug withdrawal in juvenile patients (*p* < 0.05) (Table 4).

**Table 4:** Results of the multivariate binary logistic regression analysis for recurrence after drug discontinuation in pediatric patients with epilepsy.

Factor	Regression coefficient	Standard error	Wald *χ*^2^ value	*p*-value	OR-value	95%CI of OR
History of febrile seizures	1.464	0.628	5.430	0.020	4.322	1.262–14.804
Combined ASM treatment	1.565	0.624	6.299	0.012	4.783	1.409–16.238
EEG abnormalities after drug withdrawal	1.545	0.715	4.666	0.031	4.688	1.154–19.050
Constant	−2.857	0.646	19.556	0.000	0.057	

## 4. Discussion

Epilepsy is a chronic brain disease with various causes. The incidence of epilepsy is significantly higher in people of extreme ages (14). Currently, ASMs are still the mainstay treatment for epilepsy. However, the standard for when the ASMs should be reduced or how long the reduction period should last is unclear. In 1994, researchers have proposed that tapering can start if the patient is seizure-free for ≥2 years (15). Since then, most clinicians have increasingly adopted this stance (9). However, another view stated that starting a gradual reduction in drugs after at least 3 years without a seizure can help reduce the recurrence rate (13). Fisher et al. (1) believe that the longer the remission period before drug withdrawal, the lower the risk of relapse. Recent studies by the American Academy of Neurology showed probable lack of significant difference in seizure recurrence between those who begin tapering ASMs after 2 years and those after 4 years of being seizure-free (16). How long it takes from a decision to start tapering to stopping is another issue that remains unclear. The Italian League Against Epilepsy guidelines recommend slow tapering for several weeks to several months and the specific duration should be based on the evaluation of the actual condition of the patient (17). In the case of combination medication, the drug should be gradually tapered and then discontinued. Only one drug can be tapered each time, and the time of each reduction should not be <3 months. For monotherapy patients, the tapering period should be >6 months (18). With the continuous development of ASMs, seizure control is increasingly getting better and relapse after drug withdrawal has become a focus of concern. Verrotti et al. (11) analyzed pediatric patients who were seizure-free for 2 years, and the percentage of patients with recurrence after drug withdrawal was 28.6%. Eighty patients who had been seizure-free for ≥2 years and who had normal EEG findings before drug cessation were included herein. In the 2-year follow-up period, 19 patients had a relapse, and the recurrence rate was 23.75%. It took an average of 11.09 ± 7.57 months for a recurrence. Moreover, 2 (4.9%) of the 41 patients in the nonrecurrence group had a recurrence in the third year of follow-up. Among the remaining 39 patients in the non-relapse group, 24 were followed-up until the fourth year, and no recurrence occurred. Thirteen patients were followed-up for >4 years, and no recurrence occurred. The results show that the recurrence rate is at its peak 2 years following drug cessation. This result is consistent with the findings of a previous study by Verrotti et al. (11). EEG anomalies preceding drug cessation have significant importance in predicting recurrence (19–21). As an independent risk factor for recurrence following drug withdrawal, the recurrence incidence was much greater in pediatric patients with pre-withdrawal EEG abnormalities than in individuals with normal EEG (12, 22). The recurrence rate herein was slightly lower than the 28.6% rate reported by Verrotti A et al., which may be due to normal pre-withdrawal EEG in the included pediatric patients. This study did not evaluate epileptic syndrome. Previous authors have explored ASM withdrawal in specific epilepsy syndromes, with a recurrence rate ranging from <20% (i.e., childhood absence epilepsy, childhood occipital visual epilepsy) to >80% (e.g., epilepsy with eyelid myoclonia and juvenile myoclonic epilepsy). Hence, the low relapse rate found in the study cohort may also be explained by a high prevalence of more benign syndromes (23–26).

In addition to the abovementioned timing and speed of drug reduction, other factors of recurrence risk after drug withdrawal in pediatric patients with epilepsy include sex, age at disease onset, family history, history of febrile seizures, etiology, pretreatment EEG, pretreatment disease course, epilepsy duration before remission, ASM type, age at drug withdrawal, seizure type, and EEG before and after tapering (11, 27). Currently, no international consensus has been established on the correlation between some risk factors and epilepsy recurrence. According to this study, no significant differences were found between the two groups in terms of sex, family history, age at onset, frequency of pretreatment seizures, pretreatment illness course, age at drug withdrawal, seizure type, and pretreatment EEG results (*p* > 0.05) (Table 2). Most studies have shown that sex had no significant effect on recurrence (28–31), but some researchers have found that women were more susceptible to recurrence after drug withdrawal than men (32–35). There are conflicting opinions about family history as well. A previous study revealed that pediatric patients with a positive family history have a greater recurrence rate following drug withdrawal (36). However, some researchers have discovered a lack of connection between them (32). Currently, studies have shown that a positive family history can reduce the recurrence rate of epilepsy, which may be because children with a positive family history mostly had idiopathic epilepsy without organic lesions in the brain. As for the relationship between the etiology and recurrence of epilepsy, previous research results clearly show that children with epilepsy with obvious structural damage, such as congenital brain development abnormalities, obvious organic nerve damage, brain tumors, and other definite imaging abnormalities, have a higher risk of recurrence after drug withdrawal than children with epilepsy with unknown causes (37, 38). The Italian Anti-epileptic League guidelines recommend that ASM should not be discontinued in children with imaging abnormalities (17). Herein, all the children had no obvious imaging abnormalities, 12 of them had family history, which was considered related to genetic factors. The remaining 68 cases responded well to antiseizure medication therapy; therefore, most of the children did not undergo genetic testing, and the etiology was considered as epilepsy of unknown cause. Among the 12 children with genetic factors, 4 cases had recurrence (33.3%); among 68 children with unknown cause, 15 cases had recurrence (22.1%). Herein, the recurrence rates in patients with an age of onset of 0–3 and ≥ 4 years were 27.7 and 19.2%, respectively, which were higher than the recurrence rate (14.3%) in patients aged 3–4 years (Table 3). The age of onset and time of epilepsy recurrence exhibit a U-shaped trend, according to Lamberink et al. (36) and Andersson et al. (19). The recurrence risk is the highest at birth and the lowest at age 3–4 years, gradually increases with age until 10 years, reaches a plateau at 10–25 years and increases with age after 25 years. The results of this study revealed a trend consistent with that observed in previous studies. We did not find a statistically significant relationship between the age of onset and epilepsy recurrence, which was reported in most of the previous studies (34, 35); this difference may be related to the small sample size and short follow-up duration. Cardoso et al. (39) found that a pretreatment seizure frequency of >10 is an important risk factor for recurrence after drug withdrawal. This study showed no statistically significant difference in the frequency of pretreatment seizures between the relapsed and non-relapse groups (*p* > 0.05). No pattern was found between the number of seizures before treatment and the time of recurrence in children who relapsed (Figure 2B). Other researchers have found that the recurrence rate was higher when treatment was started 6 months after epileptic seizures (31). However, the results of this study revealed no significant distinction in disease duration before treatment between the two groups. Similarly, no pattern was found in the scatter plot of the pretreatment disease course and time of recurrence (Figure 2A). The longer the epilepsy duration before remission, the higher the risk of relapse after drug withdrawal (40). Among the risk factors for relapse in children with epilepsy, seizure type is considered a relatively important recurrence factor (41). At present, the recurrence rate of focal seizures is higher than that of generalized seizures (42); however, different studies have reported results that the seizure type is not a factor affecting recurrence but the number of seizure types. Children with multiple seizure types have a higher recurrence rate than children with a single seizure type (41, 43). In the present study, there was no statistically significant between seizure type and recurrence, although this finding should be interpreted with the consideration of certain limitations. Among these are the small number of included cases, the failure of some parents to observe clinical manifestations at the time of seizure onset, and the failure to record seizures origin during EEG monitoring in most cases, increasing the number of children with generalized seizures during follow-up.

Compared with the nonrecurrence group, the recurrence group included substantially more pediatric patients with a history of febrile seizures, combined use of ≥2 ASMs, and EEG abnormalities following drug withdrawal (*p* < 0.05). The results of a bivariate logistic regression analysis revealed that EEG abnormalities following drug withdrawal, concomitant ASM therapy, and a history of febrile seizures were independent risk factors for recurrence following drug withdrawal (p < 0.05) (Table 4).

According to some studies, epilepsy that requires the combined use of ≥2 ASMs before drug withdrawal for control represents disease severity (44). For epilepsy types that can be controlled by monotherapy, the risk of relapse after drug cessation is significantly lower than the type that requires ≥2 drugs to control (40). The pediatric patients included in this study had normal EEG results before tapering to complete withdrawal. However, 6 of the 11 individuals who experienced EEG abnormalities after drug cessation had a relapse, and the difference between the two groups was significant. Currently, some studies have found that valproic acid and other ASMs can significantly inhibit epileptiform discharge in neurons (45). Thus, normal EEG results during drug reduction or withdrawal do not fully indicate complete remission. The importance of epileptiform discharge during tapering and after complete drug withdrawal in predicting relapse is gradually being recognized (45, 46). Therefore, tapering should be stopped when epileptiform discharge occurs in EEG during tapering. After drug withdrawal, EEG should be used to determine whether ASMs should be continued if there is a major anomaly.

In summary, based on previous clinical treatment experience and the content of this study, we observed that drug tapering began when there was no seizure for at least 2 years and the EEG returned to normal, and the duration of drug tapering was longer than 6 months, which could reduce the recurrence rate to some extent. To avoid recurrence during drug withdrawal, EEG should be conducted frequently and assessed during tapering. Due to the limited number of patient samples herein, the results had some limitations, which may affect the accuracy of the results. Herein, compared with the nonrecurrence group, the recurrence group had substantially more pediatric patients who had a history of febrile seizures, combined use of ≥2 ASMs, and EEG abnormalities following drug cessation. Most recurrences were observed in the first 2 years following drug cessation, and the recurrence rate was minimal thereafter. Some of the risk variables described in this study are still a subject of disagreement among international experts. Most studies on drug withdrawal in pediatric patients with epilepsy were retrospective ones, had a small sample size, or were uncontrolled studies. Thus, prospective, sizable, and randomized controlled studies are needed. In the future, in-depth studies can guide drug tapering in pediatric patients with epilepsy.

## Data availability statement

The raw data supporting the conclusions of this article will be made available by the authors, without undue reservation.

## Ethics statement

The studies involving human participants were reviewed and approved by the Medical Ethics Committee of Qilu Hospital of Shandong University. Written informed consent to participate in this study was provided by the participants’ legal guardian/next of kin.

## Author contributions

YZ and BL were responsible for the overall study design and writing the manuscript. YZ and XZ were responsible for recording patient follow-up and collecting clinical data. HD and XQ were responsible for the statistical analysis of the data. All the authors have read and approved the final manuscript.

## Funding

This study was supported by the Foundation of the National Key Research and Development Program of China (No. 2016YFC1306202).

## Conflict of interest

The authors declare that the research was conducted in the absence of any commercial or financial relationships that could be construed as a potential conflict of interest.

## Publisher’s note

All claims expressed in this article are solely those of the authors and do not necessarily represent those of their affiliated organizations, or those of the publisher, the editors and the reviewers. Any product that may be evaluated in this article, or claim that may be made by its manufacturer, is not guaranteed or endorsed by the publisher.
